# Somatotype and bioelectrical impedance vector analysis of Italian CrossFit® practitioners

**DOI:** 10.1016/j.heliyon.2024.e29139

**Published:** 2024-04-15

**Authors:** Álex Cebrián-Ponce, Sofia Serafini, Cristian Petri, Marta Carrasco-Marginet, Pascal Izzicupo, Gabriele Mascherini

**Affiliations:** a INEFC-Barcelona Sports Sciences Research Group, Institut Nacional d’Educació Física de Catalunya (INEFC), University of Barcelona (UB), 08038, Barcelona, Spain; b Department of Medicine and Aging Sciences, University “G. D'Annunzio” of Chieti-Pescara, 66100, Chieti, Italy; c Department of Sports and Computer Science, Section of Physical Education and Sports, Universidad Pablo de Olavide, 41013, Seville, Spain; d Exercise Science Laboratory Applied to Medicine “Mario Marella”, Department of Experimental and Clinical Medicine, University of Florence, 50134, Florence, Italy

**Keywords:** Phase angle, Sport, Body composition, Phantom, BIVA, Functional training

## Abstract

**Objectives:**

CrossFit® is a high-intensity sport characterized by various workouts that require strength, speed, endurance, or agility, impacting participants' body composition. This observational study aimed to determine the morphological (anthropometrical and bioelectrical) profile of CrossFit® athletes and to compare them with other athletic populations.

**Methods:**

Anthropometrical measurements and bioelectrical vector analysis (classic and specific approaches) were performed on 145 CrossFit® practitioners (107 men aged 30.7 ± 8.4 years and 38 women aged 28.1 ± 6.7 years). Each participant's relative somatotype was calculated and compared between sexes and with a Spanish CrossFit® athletes' group. Resistance-reactance graphs and Hotelling's T2 test were applied to characterize the sample, compare them with an athletes' reference population, and identify differences between somatotype groups.

**Results:**

The most represented somatotype in both groups was the balanced mesomorph (male 3.5–5.2 - 1.7 and female 4.4–4.5 - 1.8). Compared with Spanish CrossFit® athletes, significant differences were denoted for men but not women (SAD = 2.3). The bioelectrical graphs indicated that the distribution of CrossFit® athletes is quite heterogeneous and within average values for the athlete's reference. The mesomorphic and endomorphic components were associated with a higher phase angle.

**Conclusions:**

CrossFit® practitioners predominantly present a mesomorphic component and show a body type like other power athletes, although with less pronounced characteristics. The somatotype may influence the vector's position in the RXc graphs. This study provided the bioelectrical tolerance ellipses for CrossFit® practitioners in classic and specific approaches for the first time.

## Highlights

• This study shows that CrossFit® practitioners mostly have a mesomorphic somatotype, offering a solid reference for practitioners and scholars in this discipline.
• The tolerance ellipse references from the present study are helpful for comparing with athletes from this or other sports.
• The tolerance ellipse references from the present study can be used to evaluate changes produced by training or nutritional strategies and adjust them to reach their optimal level.

## List of abbreviations

BC: body composition
BIVA: bioelectrical impedance vector analysis
BM: body mass
BMI: body mass index
ECW: extracellular water
FM: fat mass
FFM: fat free mass
ICW: intracellular water
ISAK: International Society of the Advancement of Kinanthropometry
PhA: phase angle
TBW: total body water
R: resistance
Rsp: Specific resistance
SAD: somatotype attitudinal distance
Xc: reactance
Xcsp: specific reactance
Z: impedance/vector length
Zp-scores: Phantom Z-scores
Zsp: specific impedance

## 1 Introduction

CrossFit® stands out as a sport that has witnessed substantial growth in athlete participation over the past two decades [1]. Characterized by high-intensity regimens integrating weightlifting, calisthenics, and cardiovascular exercises, its workouts exhibit notable variability due to the comprehensive array of exercises involved [2]. The foundation of CrossFit® revolves around consistently varying functional movements at high intensity to enhance fitness and promote health [3]. Such variability provides physical adaptions in the different manifestations of physical exercise with the corresponding changes in body composition (BC).

For example, recreationally active women who started practicing CrossFit® significantly improved BC regarding mass (FM) and bone mass, after 16 weeks of training [4]. Furthermore, previously inactive overweight men improved body composition (weight, body mass index [BMI], body fat percent), beyond physiological parameters (resting heart rate, diastolic blood pressure, maximum oxygen uptake, peak and average power), and blood lipids after four weeks of CrossFit® training [5]. Regarding athletes and practitioners with regular practice, CrossFit® athletes show lower adiposity compared to physically active controls matched for age and BMI. Furthermore, whole-body bone mineral density, lean soft mass, and %FM differ among CrossFit® athletes according to the amount of training [6]. On the other hand, an 8-weeks Mediterranean Diet plan did not affect BC in well trained CrossFit® athletes/practitioners [7], although it may positively affect CrossFit® performance.

Among the methods developed to assess BC, anthropometry is a science that studies size, shape, proportionality, composition, and bodily function in order to understand the processes involved in exercise, nutrition, and sports performance [2]. A recent study including well-trained CrossFit® athletes showed that muscle mass positively correlates with total CrossFit® results [2]. In such a study, athletes showed low FM (11.60 % and 15.23 % for men and women, respectively) and a small relative size, which can benefit bodyweight exercise and a high muscle mass. The average somatotype (mesomorphy – endomorphy - ectomorphy) was 2.0–6.8–1.1 and 2.6–4.9 - 1.9 for men and women, respectively, denoting the prevalence of the muscular component associated with low FM.

An alternative method to assess BC is the bioelectrical impedance vector analysis (BIVA), which consists of evaluating the raw bioelectrical resistance (R) and the reactance (Xc) parameters, and its derived components, the impedance/vector length (Z) and the phase angle (PhA) [8]. When applied to different populations, this method overcomes the potential inaccuracy of predictive equations because it compares the vector position to the tolerance ellipses representing the population reference values without elaboration [9]. Two BIVA approaches differ according to the standardization of the bioelectrical parameters [10]. In the classic BIVA approach, adjustments are made for stature to reduce the effect of conductor length, representing a valid method for assessing body fluids. In contrast, in the specific BIVA approach, adjustments are made for stature and cross-sectional areas (arms, trunk, and legs) to reduce the effect of body volume, representing the percentage of fat mass (%FM) [11,12]. Therefore, the height-adjusted vector length (Z/H) is inversely related to total body water (TBW) [8], and specific vector length (Zsp) is positively correlated with %FM [13]. PhA can be considered a marker of cellular health and integrity of cell membranes. In addition, showed an inverse correlation with the ratio of extracellular to intracellular water (ECW/ICW), independently of the BIVA approach [14]. BIVA has been studied in many sports [15], plotting the athletes within a tolerance ellipse of a given reference population, performing intra- or inter-group comparisons, monitoring athletes through a competitive season [16,17], or creating new tolerance ellipses for specific sports population, as it has been made in soccer players and referees [18,19], cyclists [20], synchronized swimmers [21], handball players [22] or natural bodybuilders [23]. To our knowledge, this is the first study analyzing the complete bioelectrical profile in CrossFit®. Only Campa, Thomas et al. [24] provided reference percentiles of PhA values in CrossFit®, along with many other athletes of different sports.

Thus, the present study aimed to characterize the morphology of a sample of CrossFit® practitioners, according to the anthropometrical and the BIVA methods (classic and specific approaches), and to compare them with other athletic samples in the literature. Furthermore, this study also aimed to provide specific positioning within the R-Xc graph, which can contribute increasing the knowledge of the BC of these athletes. We hypothesized that the current sample would show a typical somatotype, with the balanced mesomorph as the most represented, followed by endomorph-mesomorph. Furthermore, these athletes should be comparable to the athletic population practicing power and strength sports disciplines [14,24], and bioelectrical differences among somatotype groups are expected.

## 2 Material and methods

### 2.1 Subjects

This observational study involved 145 Italians CrossFit® practitioners, 107 men (age: 30.7 ± 8.4 years; BM: 81.9 ± 14.7 kg; stature: 177.3 ± 6.0 cm) and 38 women (age: 28.1 ± 6.7 years; BM: 65.5 ± 10.1 kg; stature: 166.2 ± 6.3 cm). The eligibility criteria encompassed individuals meeting the following conditions: (1) being highly trained, defined as engaging in physical activity for a minimum of 10 h per week, comprising five sessions of approximately 2 h each, involving a combination of endurance and resistance exercises, or having achieved national-level competition status; (2) absence of injuries or any clinical conditions at the time of enrollment; (3) refraining from pharmacological therapy or medication intake within the preceding 48 h; (4) for female participants, being in a postmenarcheal state; (5) not undergoing pharmacological regulation for contraception or menstrual cycle control. All athletes participated voluntarily, having been thoroughly briefed on the entire process and methodology employed, and subsequently providing written consent in accordance with the principles outlined in the Declaration of Helsinki 2013. The study was approved by the Ethics Committee for Clinical Sports Research of Catalonia (Ethical Approval Code: 0022/CEICGC/2023).

### 2.2 Procedures

This study was conducted with a sample of CrossFit® athletes from the same gym. Participant recruitment occurred at the start of the season, and data collection for the study was completed within the following month. All the anthropometric and bioelectrical evaluations were taken in the morning at rest, in fasting conditions, and before the first training session of the day. The day before BIA measurements, participants abstained from caffeine, alcohol, and exercise while maintaining their regular dietary regimen.

#### 2.2.1 Anthropometric measurements

All anthropometric measurements were performed according to the International Society of the Advancement of Kinanthropometry (ISAK) protocol [25] by a level 2 anthropometrism with a certified intra-tester target % technical error of measurement lower than 7.5 % for skinfolds and 1.5 % for other measures. During the entire procedures, athletes wore light clothing. Stature and body mass (BM) were assessed to the nearest 0.1 cm and 0.1 kg using a stadiometer with a balance-beam scale (Seca 200, Seca, Hamburg, Germany). Ten skinfolds, five girths, and three breadths were taken on the right side of the body. Skinfold thickness was measured to the nearest 0.2 mm using a skinfold caliper (Holstein Tanner/Whitehouse, Crosswell, Crymych, Pembs., SA41 3UF, UK). Girths were measured to the nearest 0.1 cm using a flexible anthropometric steel tape (Cescorf, Porto Alegre, Brazil). The breadths were measured to the nearest 0.1 cm using a bone caliper (Cescorf, Porto Alegre, Brazil). Utilizing a rotational approach, measurements were taken twice at each site, with a third measure added in instances where the difference exceeded 5 % for skinfold measurements and 1 % for other measures. The sum of triceps, subscapular, supraspinal, abdominal, thigh, and calf skinfolds was performed to obtain the sum of six skinfolds. Somatotype was calculated following the methods of Carter & Heath [26]. Subsequently, they were classified into seven larger groups [27] (i.e., Central type, Endomorph, Endomorph-mesomorph, Mesomorph, Mesomorph-ectomorph, Ectomorph, Ectomorph-endomorph) for the 13 somatotype categories previously defined by Carter and Heath.

#### 2.2.2 Bioelectrical measurements

R and Xc were assessed using a BIA 101 Anniversary Sport Edition analyzer (Akern Srl, Florence, Italy) that emitted a 400 mA alternating sinusoidal current at 50 kHz (±0.1 %). To unsure the device calibration, a known impedance circuit provided by the manufacturer was utilized. The impedance values were R = 383 ± 10 Ω and Xc = 45 ± 5 Ω and a coefficient of variation of 0.2.

According to the manufacturer guidelines, participants were tested with their arms and legs kept from touching the body by non-conductor foam objects to prevent adduction or the crossing of the limbs. After a stabilization period of 5 min, bioelectrical measurements were recorded. During the procedure participants remained motionless to ensure the proper distribution of body fluids. Injector electrodes (Biatrodes Akern Srl, Firenze, Italia) were placed on the dorsal surface of the right hand (proximal to the third metacarpal-phalangeal joint) and foot (proximal to the third metatarsal-phalangeal joint). To mitigate electric field interaction, the detector electrodes were positioned approximately 5 cm away from the injector, potentially reducing the risk of overestimating impedance values.

Z was calculated as (R^2^ + Xc^2^)^0.5^, and PhA as tan−1 (Xc/R · 180°/π). R, Xc, and Z were adjusted by stature for the classic BIVA approach (R/H, Xc/H, Z/H) and by the stature and cross-sectional areas of the arm, trunk, and leg for the specific BIVA approach (Rsp, Xcsp, Zsp). RXc point graphs were utilized to plot the points of the CrossFit® practitioners regarding the 50 %, 75 %, and 95 % classic and specific tolerance ellipses of the reference population [14], which consisted of 139 male athletes (age: 21.5 ± 5.0 years; BM: 77.2 ± 11.4 kg; stature: 183.3 ± 9.1 cm) and 63 female athletes (age: 20.7 ± 5.1 years; BM: 63.7 ± 8.9 kg; stature: 171.1 ± 8.2 cm) of 11 different sports (athletics, basketball, handball, judo, karate, modern pentathlon, rugby, soccer, swimming, triathlon, volleyball). Fat-free mass (FFM), TBW, and ECW were obtained by applying the formulas developed by Matias et al. [28,29]. FM, ICW, and ECW/ICW ratio were calculated from previous values.

### 2.3 Statistical analysis

The normality of the data distribution was verified using the Shapiro–Wilk test, and descriptive statistics were performed for each variable, as reported in Table 1, Table 2. All variables followed the Gaussian distribution. Each participant was plotted into the somatochart, The chi-square test was used for comparison of the frequency of different somatotype categories between men and women. The somatotype attitudinal distance (SAD) for male and female practitioners was calculated for comparison with Spanish CrossFit® athletes [2] and inter-group comparison (i.e., men Vs. women). A SAD ≥2 was considered as a distance statistically different between two somatotype means [26]. The anthropometric values for each CrossFit® practitioner were normalized against the Phantom stature (170.18 cm), and their anthropometric profiles were compared to the Phantom model (via Z-scores calculation) to examine differences in proportionality between men and women [30,31]. If possible, all data were presented as mean ± standard deviation, apart from Phantom Z-scores (Zp-scores) expressed as means ± standard errors of the mean according to de Ridder, Smith, Wilders, and Underhay [32]. The student's independent sample *t*-test was used to compare Zp-scores between men and women. An RXc point graph and two-sample Hotelling's T2 test were used to characterize and identify BIVA differences in the sample compared to the athletic reference population [14]. A minimum total sample size of 102 participants was calculated through an a priori power analysis (using G*Power 3.1, Heinrich-Heine-Universität, Düsseldorf, Germany) with an 80 % power and a significance level (alpha) of 0.05 and a specified effect size of 0.5. Statistical calculations were performed using SPSS (version 21, Chicago, IL, USA), while BIVA software [33] was utilized for graphing, comparing bioelectrical parameters, and computing tolerance ellipses (50 %, 75 %, and 95 %) for the study sample. Significance was determined with a threshold of p < 0.05.

**Table 1:** Anthropometric and somatotype components of the CrossFit® practitioners.

		Meso	Endo-Meso	Endo	Ecto	Meso-Ecto	Central type	Total
**Stature (cm)**	M	176.4 ± 6.1	176.5 ± 4.1	178.3 ± 4.8	181.9 ± 5.7	181.0 ± 2.0	182.3 ± 7.1	177.3 ± 6.0
	F	165.8 ± 6.6	167.7 ± 4.8	164.6 ± 4.6	183.0	–	167.0 ± 9.0	166.2 ± 6.3
**BM (kg)**	M	82.5 ± 14.3	87.4 ± 12.6	90.8 ± 17.6	65.1 ± 4.2	70.5 ± 3.7	82.2 ± 12.4	81.9 ± 14.7
	F	66.4 ± 13.4	64.9 ± 5.3	66.3 ± 7.9	69.2	–	57.8 ± 8.3	65.5 ± 10.1
**BMI (kg/m**^**2**^**)**	M	26.4 ± 3.7	28.0 ± 2.9	28.4 ± 4.4	19.7 ± 1.1	21.5 ± 0.7	24.6 ± 2.4	26.0 ± 4.0
	F	24.0 ± 3.6	23.1 ± 0.8	24.4 ± 2.2	20.7	–	20.6 ± 0.8	23.6 ± 2.8
**Σ6 skinfolds (mm)**	M	74.5 ± 31.6	130.5 ± 31.3	137.1 ± 43.0	45 ± 13.5	38.3 ± 2.9	66.7 ± 7.5	81.2 ± 40.0
	F	78.3 ± 32.1	91.4 ± 11.5	126.0 ± 31.8	63.6	–	76.7 ± 2.4	96.2 ± 35.2
**Endomorphy**	M	3.2 ± 1.5	5.6 ± 1.2	6.2 ± 1.5	1.7 ± 0.5	1.4 ± 0.3	2.5 ± 0.5	3.5 ± 1.8
	F	3.5 ± 1.5	4.4 ± 0.5	5.7 ± 1.3	3.0	–	3.1 ± 0.4	4.4 ± 1.6
**Mesomorphy**	M	5.7 ± 1.4	5.6 ± 1.2	5.2 ± 1.5	2.1 ± 0.8	3.5 ± 0.3	2.8 ± 1.0	5.2 ± 1.7
	F	5.1 ± 1.5	4.2 ± 0.5	4.5 ± 1.1	2.2	–	3.0 ± 0.3	4.5 ± 1.4
**Ectomorphy**	M	1.5 ± 0.8	0.9 ± 0.6	1.1 ± 0.9	4.6 ± 0.8	3.6 ± 0.2	2.2 ± 0.8	1.7 ± 1.2
	F	1.7 ± 0.8	2.0 ± 0.4	1.4 ± 0.7	4.1	–	3.1 ± 0.2	1.8 ± 0.9

Data are presented as mean ± standard deviation; BM = body mass, BMI = body mass index, Ecto = balanced ectomorph, Endo = balanced endomorph, Endo-Meso = endomorph-mesomorph, Meso = balanced mesomorph, Meso-Ecto = mesomorph-ectomorph, F = females; females: mesomorph (n = 15), endomorph-mesomorph (n = 6), endomorph (n = 13), ectomorph (n = 1), central type (n = 3); M = males, males: mesomorph (n = 73), endomorph-mesomorph (n = 11), endomorph (n = 8), ectomorph (n = 9), mesomorph-ectomorph (n = 3); central type (n = 3).

**Table 2:** Bioelectrical data of the CrossFit® practitioners.

		Meso	Endo-Meso	Endo	Ecto	Meso-Ecto	Central type	Total
**R/H (Ω/m)**	M	246.6 ± 31.9	271.7 ± 20.9	275.4 ± 37.5	280.8 ± 19.4	265.2 ± 28.1	244.3 ± 37.4	254.6 ± 32.7
	F	317.7 ± 48.5	351.1 ± 26.0	354.4 ± 30.0	345.4	–	345.3 ± 21.4	338.4 ± 39.9
**Xc/H (Ω/m)**	M	33.4 ± 4.7	35.0 ± 3.0	36.3 ± 5.7	35.1 ± 3.3	33.5 ± 4.5	32.4 ± 3.9	33.9 ± 4.5
	F	36.9 ± 4.7	42.7 ± 6	40.7 ± 5.2	38.3	–	39.0 ± 2.1	39.3 ± 5.2
**Z/H (Ω/m)**	M	248.8 ± 32.1	273.9 ± 21.1	277.8 ± 37.7	283.0 ± 19.4	267.3 ± 28.4	246.4 ± 37.6	256.9 ± 32.8
	W	319.8 ± 48.6	353.7 ± 26.1	356.7 ± 30.2	347.5	–	347.5 ± 21.3	340.7 ± 40.1
**R*sp* (Ω·cm)**	M	328.1 ± 46.6	390.0 ± 62.3	390.9 ± 43.2	274.7 ± 25.4	282.3 ± 17.8	276.8 ± 39.3	331.9 ± 55.7
	F	340.6 ± 69.1	361.9 ± 37.2	382.2 ± 58.9	344.1	–	309.7 ± 28.7	355.8 ± 60.7
**X*csp* (Ω·cm)**	M	44.4 ± 6.8	50.4 ± 8.5	51.4 ± 5.2	34.5 ± 4.6	35.7 ± 3.8	37.0 ± 6.3	44.3 ± 7.9
	F	39.6 ± 7.0	43.7 ± 4.0	43.7 ± 6.3	38.1	–	34.9 ± 2.5	41.2 ± 6.4
**Z*sp* (Ω·cm)**	M	331.1 ± 46.9	393.2 ± 62.8	394.3 ± 43.3	276.8 ± 25.7	284.6 ± 18	279.3 ± 39.7	334.9 ± 56.1
	W	342.9 ± 69.3	364.6 ± 36.9	384.7 ± 59.1	346.2	–	311.7 ± 28.7	358.2 ± 60.9
**PhA (°)**	M	7.7 ± 0.8	7.3 ± 0.4	7.5 ± 0.7	7.1 ± 0.6	7.2 ± 0.4	7.6 ± 0.6	7.6 ± 0.8
	F	6.7 ± 0.7	7.0 ± 0.9	6.6 ± 0.6	6.3	–	6.5 ± 0.4	6.7 ± 0.7
**FFM (kg)**	M	70.4 ± 10.2	70.5 ± 7.5	72.5 ± 12.2	58.6 ± 2.6	62.7 ± 4.4	71.2 ± 10.8	69.4 ± 10.1
	F	50.1 ± 9.1	47.5 ± 3.3	47.8 ± 4.6	51.4	–	43.9 ± 5.8	48.5 ± 6.7
**FM (kg)**	M	12.2 ± 5.1	17.0 ± 5.6	18.4 ± 5.9	6.5 ± 2.2	7.8 ± 0.9	11.0 ± 1.7	12.5 ± 5.6
	F	16.3 ± 5.2	17.4 ± 2.5	18.5 ± 3.6	17.8	–	13.8 ± 2.7	17.1 ± 4.2
**FM (%)**	M	14.4 ± 3.7	19.0 ± 3.7	19.9 ± 2.8	9.8 ± 2.8	11.1 ± 1.8	13.4 ± 0.0	14.8 ± 4.2
	F	24.4 ± 3.5	26.7 ± 2.0	27.7 ± 2.8	25.7		23.8 ± 2.0	25.9 ± 3.2
**TBW (L)**	M	51.4 ± 7.1	51.8 ± 5.4	53.2 ± 8.5	43.1 ± 1.8	45.9 ± 2.9	51.8 ± 7.3	50.7 ± 7.0
	F	36.3 ± 6.4	34.6 ± 2.3	34.9 ± 3.3	37.3	–	32.0 ± 4.0	35.2 ± 4.7
**TBW (%)**	M	62.6 ± 2.6	59.5 ± 2.7	58.9 ± 2.2	66.3 ± 1.9	65.1 ± 0.9	63.1 ± 0.7	62.4 ± 3.0
	F	54.8 ± 2.1	53.4 ± 1.3	52.8 ± 1.8	53.8		55.5 ± 1.5	53.9 ± 2.0
**ECW (L)**	M	16.2 ± 2.2	16.7 ± 1.7	17.1 ± 2.7	13.9 ± 0.6	14.7 ± 0.8	16.4 ± 2.0	16.1 ± 2.2
	F	12.8 ± 2.0	12.2 ± 1.0	12.4 ± 1.2	13.2	–	11.5 ± 1.3	12.5 ± 1.5
**ICW (L)**	M	35.1 ± 5.0	35.1 ± 3.7	36.1 ± 5.9	29.2 ± 1.4	31.2 ± 2.1	35.5 ± 5.3	34.6 ± 4.9
	F	23.5 ± 4.4	22.4 ± 1.4	22.5 ± 2.2	24.0	–	20.5 ± 2.7	22.8 ± 3.2
**ECW/ICW ratio**	M	0.46 ± 0.02	0.48 ± 0.01	0.47 ± 0.02	0.48 ± 0.02	0.47 ± 0.01	0.46 ± 0.02	0.47 ± 0.02
	F	0.55 ± 0.03	0.54 ± 0.02	0.55 ± 0.02	0.55		0.56 ± 0.02	0.55 ± 0.02

Data are presented as mean ± standard deviation; Ecto = balanced ectomorph, ECW = extracellular water, Endo = balanced endomorph, Endo-Meso = endomorph-mesomorph, FFM = fat free mass, FM = fat mass, ICW = intracellular water, M = males, Meso = balanced mesomorph, Meso-Ecto = mesomorph-ectomorph, PhA = phase angle, R/H = height-adjusted resistance, R*sp* = specific resistance, TBW = total body water, F = females, Xc/H = height-adjusted reactance, Xc*sp* = specific reactance, Z/H = height-adjusted vector length, Z*sp* = specific vector length; Females: mesomorph (n = 15), endomorph-mesomorph (n = 6), endomorph (n = 13), ectomorph (n = 1), central type(n = 3); Males: mesomorph (n = 73), endomorph-mesomorph (n = 11), endomorph (n = 8), ectomorph (n = 9), mesomorph-ectomorph (n = 3); central type (n = 3).

## 3 Results

Anthropometric characteristics and somatotype components of the CrossFit® practitioners are represented in Table 1. The sample is divided according to sex and the seven previously described somatotype categories. The complementary information (skinfolds, girths, and breadths) can be observed in.

**Fig. 1:**
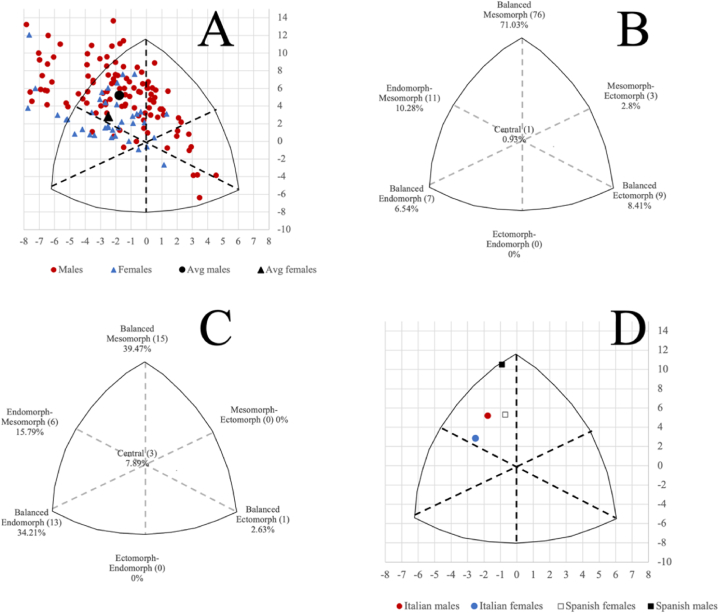
A, somatotype distribution of male and female CrossFit® practitioners. The small indicators represent the individual values, and the large ones, the mean values; B, somatotype categories of male CrossFit® practitioners; C, somatotype categories of female CrossFit® practitioners; D, somatotype distribution of Spanish [2] and Italian male and female CrossFit® practitioners.

The average somatotype (mesomorphy – endomorphy - ectomorphy) for male and female CrossFit® practitioners was 3.5–5.2–1.7 and 4.4–4.5 - 1.8, respectively (Fig. 1A). In men, the prevalent somatotype was balanced mesomorph, followed by endomorph-mesomorph, balanced ectomorph, balanced endomorph, and mesomorph-ectomorph and central somatotypes, respectively (Fig. 1B). In women, the prevalent somatotype was balanced mesomorph, followed by balanced endomorph, endomorph-mesomorph, central, and balanced ectomorph somatotypes, respectively (Fig. 1C). The chi-square test indicated that the difference regarding the somatotype categories occurrence between men and women was significant (Χ^2^ = 28.086, P < 0.001). The comparison based on the SAD does not indicate significant differences between sexes (SAD = 1.11), while a significant difference was denoted for men when compared with Spanish CrossFit® athletes but not for women (SAD = 2.3 and 1.8, respectively; Fig. 1D).

**Fig. 2:**
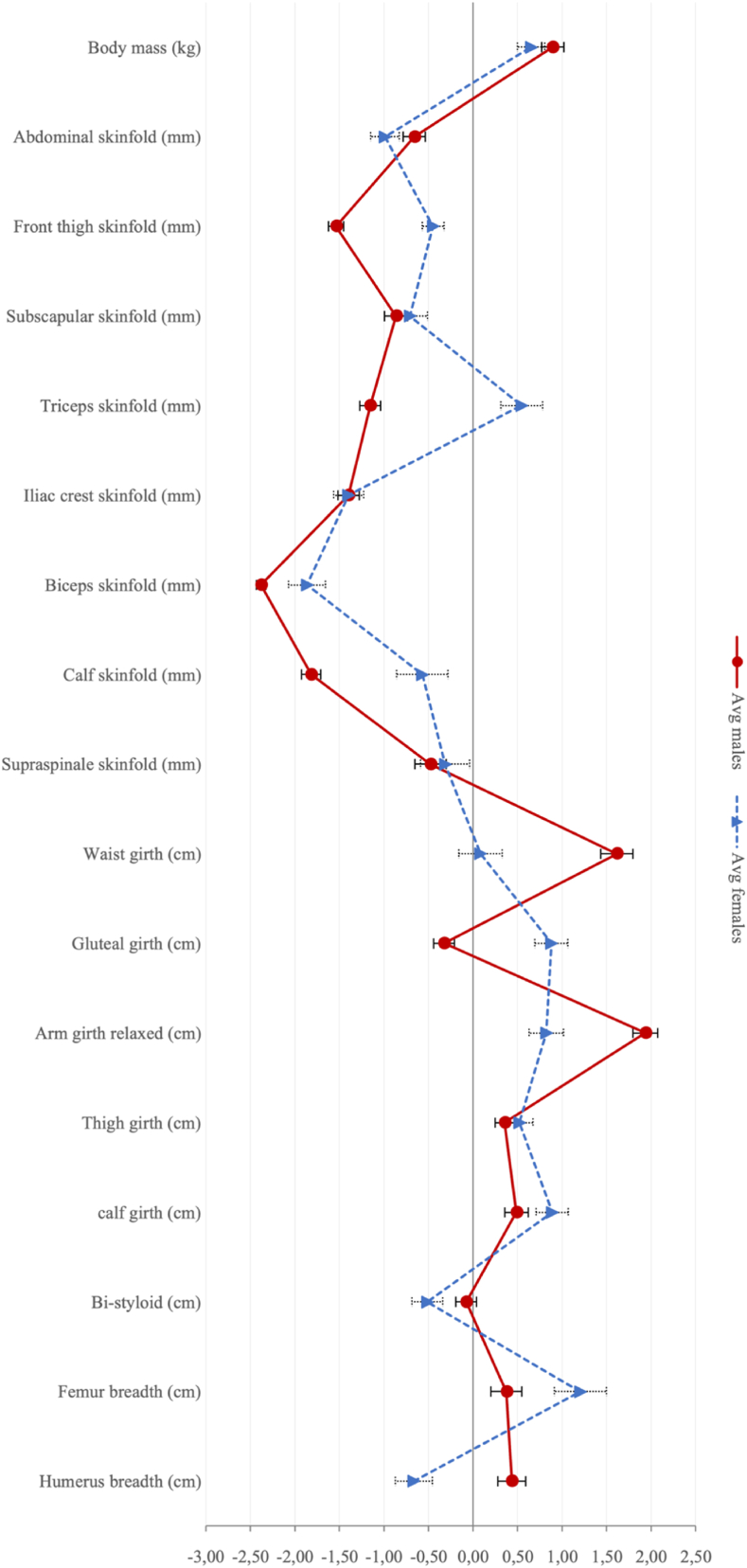
Phantom of the sample for male and female CrossFit® practitioners.

Fig. 2 shows several anthropometric dimensions of male and female CrossFit® practitioners. Relative to the Phantom, men and women have a similar pattern, with BM, girths, and diameters showing small to moderate positive Zp-scores and skinfolds negative Zp-scores. Women's triceps skinfold, males' gluteal girth, and women's styloid and humerus breadth followed an inverse trend. Men and women differ in Zp-scores of almost all anthropometric dimensions except for BM, subscapular, supra iliac, supraspinal, abdominal skinfolds, and mid-tight and leg girths.

Table 2 represents the sample's classic and specific bioelectrical characteristics, together with other BC parameters obtained from the predictive equations for athletes based on bioelectrical parameters.

**Fig. 3:**
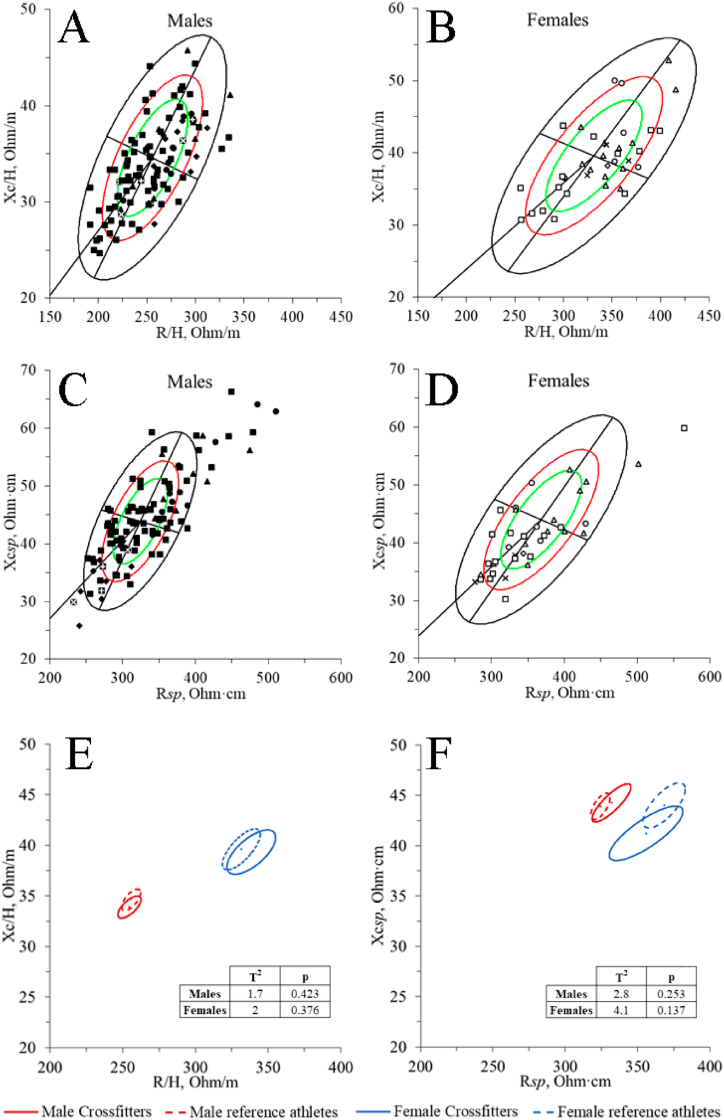
BIVA point graph of male and female CrossFit® practitioners in comparison with the athlete's reference population. A, male's classic BIVA; B, female's classic BIVA; C, male’ specific BIVA; D, female’ specific BIVA; E, classic BIVA mean graph; F, specific BIVA mean graph. ■, balanced mesomorph; ●, endomorph-mesomorph; ▲, balanced endomorph; ♦, balanced ectomorph; ✚, mesomorph-ectomorph; ☒, central type.

The classic BIVA point graphs indicate a reasonably homogeneous distribution within 95 % of the classic athletic reference ellipse in both men (Fig. 3A) and women (Fig. 3B), although in women, there seems to be a slight tendency towards the right side of the ellipse. In the specific BIVA approach, in men, again, there is a homogeneous distribution (Fig. 3C), but more practitioners fall outside 95 % of the reference ellipse by the upper right quadrant. The women are concentrated in the lower half of the ellipse (Fig. 3D). Regarding the whole-complex vector (Fig. 3E and F), there are no significant differences between CrossFit® practitioners and the athletes’ reference population, neither in men (classic: T2 = 1.7, p = 0.423; specific: T2 = 2.8, p = 0.253) nor in women (classic: T2 = 2.0, p = 0.376; specific: T2 = 4.1, p = 0.137).

**Fig. 4:**
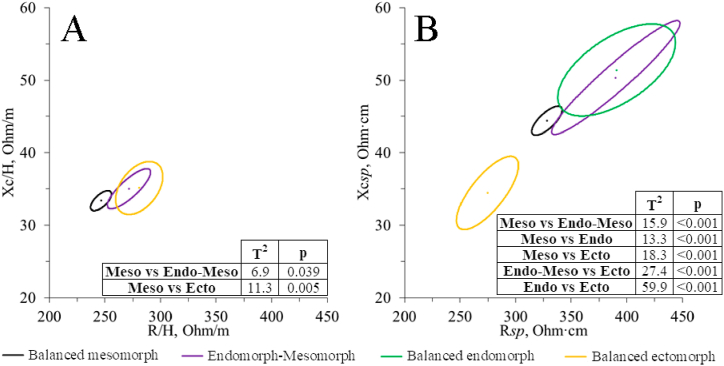
BIVA mean impedance vectors for somatotype that showed significant differences. A, men’ classic BIVA differences; B, men’ specific BIVA differences. Meso, balanced mesomorph; Endo-Meso, endomorph-mesomorph; Endo, balanced endomorph; Ecto, balanced ectomorph.

Fig. 4 compares the somatotype groups' complex vectors (only the groups with statistical differences are represented). There are significant differences in men only, both in classic (Fig. 4A) and specific BIVA (Fig. 4B). It can be seen how the balanced mesomorph athletes show a greater PhA than the rest of the groups, but the most striking differences (especially in specific BIVA) are due to the vector length.

**Fig. 5:**
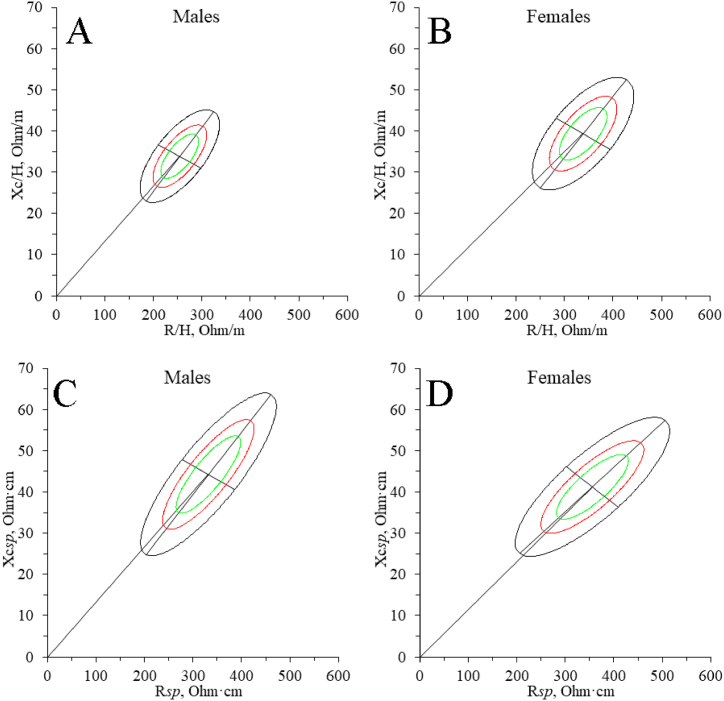
New tolerance ellipses for CrossFit® practitioners. A, men’ classic tolerance ellipse; A, men’ classic tolerance ellipse; A, females' classic tolerance ellipse; C, men’ specific tolerance ellipse; D, females' specific tolerance ellipse.

Fig. 5 represents the newly generated classic and specific tolerance ellipses of CrossFit® practitioners. In particular, Fig. 5A and B represents the male and female classic BIVA tolerance ellipses (R/H: 254.6 ± 32.7 Ω/m, Xc/H: 33.9 ± 4.5 Ω/m, r: 0.68; R/H: 338.4 ± 39.9 Ω/m, Xc/H: 39.3 ± 5.2 Ω/m, r: 0.67) respectively; Fig. 5C and D represents the male and female specific BIVA tolerance ellipses (R/H: 331.9 ± 55.7 Ω/m, Xc/H: 44.3 ± 7.9 Ω/m, r: 0.83; R/H: 355.8 ± 60.7 Ω/m, Xc/H: 41.2 ± 6.4 Ω/m, r: 0.81), respectively.

## 4 Discussion

The current study describes a sample of CrossFit® practitioners with a training routine of at least 10 h per week. The first significant result is that CrossFit® practitioners showed a predominantly mesomorph somatotype, with the endomorph component as the second major contributor to their body characteristics. Secondly, they showed bioelectrical impedance characteristics similar to those of the athletic populations [14]. Furthermore, bioelectrical differences between some somatotype groups were identified in men, especially in those with mesomorph and ectomorph components. Finally, classic and specific tolerance ellipses for this sample were developed.

CrossFit® athletes perform high-intensity efforts combining weightlifting and bodyweight fundamentals which derive mainly from gymnastics, weightlifting, powerlifting, and other sports, as well as cardiovascular exercise. For this reason, CrossFit® athletes and practitioners leverage their substantial muscle mass while minimizing FM. Even though more prominent athletes can develop higher forces, small body sizes can help during bodyweight exercises [1].

The present study reports body composition parameters in a large sample of longtime CrossFit ® practitioners for the first time; therefore, the data shown can be considered chronic adaptations to this specific discipline [34]. Previous studies highlighted the acute adaptations of a training program based on Crossfit principles for heterogeneous types of participants, both sedentary [35], physically active [36], and workers [37].

Thus, the variety of body types in CrossFit® can be large. Our sample was distributed from the right-bottom corner to the left-top corner of the somatochart, with most participants distributed on the latter site. Such a distribution denotes a large variety of body types and a prevalence of the mesomorph component, followed by the endomorph one. These morphological characteristics are typical of powerful athletes performing weightlifting, powerlifting, and artistic gymnastics [26]. However, the higher specialization of these disciplines denotes more extreme positioning on the somatochart. Indeed, while powerlifting athletes, especially heavyweights, are outside the normal range and may have abundant FM [38], gymnasts generally are curvilinear, with very low FM, to perform movement and skills against gravity efficiently and fast [39,40]. Compared to Spanish CrossFit® athletes, the current male sample is significantly different and positioned below and to the left of their Spanish counterpart [2].

Furthermore, a similar tendency can be observed in women. These differences can be ascribed to a different competitive level, considering that the current sample includes participating in competitions and not. On the other hand, differences in training routines (e.g., more strength and less cardiovascular and bodyweight activities) can determine different adaptations [6]. Indeed, the current sample shows higher FM and a bigger body size than the Spanish one.

The Phantom method indicates similar proportions for both men and women. CrossFit® athletes have larger bones and girths, with few exceptions, and thinner skin folds (Fig. 2). However, this deviation from the reference is not very large, below two Zp-scores, except for the men's biceps skinfold. Again, the current one shows less pronounced characteristics than the Spanish sample.

Regarding the difference between men and women, both the somatotype and the phantom indicate a typical sexual dysmorphism. Men have larger bones and muscle mass, and women have larger fat depots, especially on the limbs and hips.

BIVA distribution of this sample is relatively homogeneous compared to the tolerance ellipses of the reference athletic sample (Fig. 3A, B, 3C, and 3D), possibly due to the variety of body types and workout characteristics [3]. Although some trends can be observed: in the group of men, in the specific BIVA approach (Fig. 3C), numerous subjects are outside the 95 % reference tolerance ellipse. Most men outside the 95 % tolerance ellipse protrude from the upper right quadrant, indicating a higher %FM than reference. This aspect is not noticeable in the classic BIVA approach, in which only four subjects protrude from the 95 % tolerance ellipse (Fig. 3A). Therefore, significant differences between samples are due to differences in %FM rather than TBW. On the other hand, there is a tendency to plot the women towards the right quadrant for the classic BIVA approach (Fig. 3B) and towards the lower half for the specific BIVA approach (Fig. 3D), indicating a lower amount of TBW and lower %FM, respectively. Although these trends between groups are visible in the graphs, there are no significant differences between sample confidence ellipses for either classic (Fig. 3E) or specific (Fig. 3F) approaches.

In both sexes, the PhA and thus the ECW/ICW ratio, which are considered indicators of performance and cellular health [24,[41], [42], [43]], appear not to be different between samples (men: CrossFit®, 7.6 ± 0.8° Vs. athletes' reference, 7.7 ± 0.8°; women: CrossFit®, 6.7 ± 0.7° Vs. athletes’ reference, 6.8 ± 0.8°). These data are consistent with the PhA percentiles for CrossFit provided by Campa, Thomas et al. [24], which indicated that the 50th percentile for men and women is 7.7° and 6.8°, respectively. It is also very similar to the average PhA for velocity/power sports, which is 7.6° for men and 6.9° for women.

Significant bioelectrical differences were found after the division of the sample into the seven somatotype groups, especially between the balanced mesomorphs (Fig. 4A) and balanced ectomorphs (Fig. 4A and B) and the rest of the groups. In particular, the balanced mesomorph men group showed a greater PhA when compared to the endomorph-mesomorph, balanced endomorph, and balanced ectomorph groups. In women, there are no significant vector differences between groups. However, it can also be observed in Table 2 that the groups with a mesomorphic or endomorphic component have a higher PhA than the rest. This observation aligns with a previous investigation involving volleyball, rugby, and soccer players [44], which revealed that individuals with a pronounced mesomorphic component exhibited a more inclined vector and higher PhA values than athletes with a balanced ectomorphic somatotype. This association can be attributed to the correlation between elevated PhA values and greater muscle mass, as well as a higher proportion of intracellular water (ICW) relative to extracellular water (ECW) [24,[41], [42], [43]]. The aforementioned study also indicated that R/H was the first discriminant variable representing differences between athletes of different somatotypes. However, in our case, Zsp seemed more sensitive in identifying between groups since the balanced ectomorph group also differed from the endomorph-mesomorph and balanced endomorph groups by shortening the specific vector length.

The study of BC, which provides CrossFit®’s BIVA tolerance ellipse references (Fig. 5) for the first time, is helpful for comparing with athletes from this or other sports. Furthermore, these results can be used to evaluate changes produced by training or nutritional strategies and adjust them to reach their optimal level [23].

The present study has some strengths. Firstly, this is the first study providing bioelectrical vector characteristics of CrossFit® practitioners, which is important since it has been a growing sport for years [2]. Secondly, the study offers an extensive characterization of both genders employing various techniques, including anthropometry, classic BIVA, and specific BIVA. This comprehensive approach can serve as a valuable resource for researchers and practitioners alike. It is crucial to emphasize that body composition assessment can exhibit substantial variation not only in methodologies but also based on the instruments and techniques employed [9].

However, some limitations should be considered. Firstly, the study is limited by its descriptive nature, which only allows for hypothesis generation. Dividing the sample into groups according to somatotype makes the sample too small or null in some of the groups, so hypothesis tests could not be performed. Second, the study should collect information regarding previous training routines. Finally, the current sample is quite heterogeneous. This study can be helpful for general CrossFit® practitioners who need a reference. On the other hand, elite athletes may need more specific references that reflect their competitive level.

Indications for future studies that can fill the information still missing in this specific field of research could be 1) how certain specific functional training stimuli can modify the participant's positioning both within the somatochart and in the RXc points graph; 2) how the positioning of the participant within the somatochart and/or the RXc points graph corresponds to specific physical performance, strength, and endurance values.

## 5 Conclusions

In summary, this study indicated that CrossFit® practitioners, in which the mesomorphic component is predominant, show a body type like other power athletes, although with less pronounced characteristics. Furthermore, this study provided, for the first time, the BIVA ellipses for the CrossFit® practitioners, which are not significantly different from the athletic reference population. The type of somatotype may influence the position of the vector in the RXc graphs, especially in subjects with a predominant mesomorphic component by a higher PhA and in subjects with a predominant ectomorphic component by a lower %FM and, therefore, a lower Zsp.

## Funding statement

This research received no external funding.

## Data availability statement

All data generated analysed during the current study are available from Mascherini, Gabriele; Izzicupo, Pascal; Petri, Cristian (2024), “Somatotype & BIVA Crossfit”, Mendeley Data, V1, https://doi.org/10.17632/rs8zkcp6mp.1.

## CRediT authorship contribution statement

**Álex Cebrián-Ponce:** Writing – original draft, Visualization, Formal analysis, Data curation. **Sofia Serafini:** Writing – review & editing, Visualization, Formal analysis. **Cristian Petri:** Writing – review & editing, Resources, Investigation, Data curation, Conceptualization. **Marta Carrasco-Marginet:** Writing – review & editing, Validation, Methodology. **Pascal Izzicupo:** Writing – original draft, Methodology, Formal analysis, Conceptualization. **Gabriele Mascherini:** Writing – review & editing, Validation, Resources, Methodology, Investigation, Conceptualization.

## Declaration of competing interest

The authors declare that they have no known competing financial interests or personal relationships that could have appeared to influence the work reported in this paper.
